# The Gambling Factors Related with the Level of Adolescent Problem Gambler

**DOI:** 10.3390/ijerph16122110

**Published:** 2019-06-14

**Authors:** Kyonghwa Kang, Jong Sun Ok, Hyeongsu Kim, Kun-Sei Lee

**Affiliations:** 1 Department of Nursing, College of Health & Welfare & Nursing, ChungWoon University, Chungnam 32244, Korea; kh_kang@chungwoon.ac.kr; 2 Department of Nursing, College of Nursing, Konkuk University, Chungcheonbuk-do 27478, Korea; 3 Department of Preventive Medicine, School of Medicine, Konkuk University, Seoul 05029, Korea; mubul@kku.ac.kr (H.K.); leekonkuk@gmail.com (K.-S.L.)

**Keywords:** adolescent, gambling, problem gambling, prevention

## Abstract

The purpose of this study was to investigate the gambling factors related with the gambling problem level of adolescents to provide basic information for the prevention of adolescent gambling problems. The data was drawn from the 2015 Survey on Youth Gambling Problems of the Korea Center on Gambling Problems for Korean students in grades 7–11 (ages 13–17 years) and included 14,011 study subjects (average age 14.9 years, 52.5% male). The lifetime gambling behavior experience was 42.1%, and 24.2% had a gambling behavior experience within the past three months. The past three-month prevalence of problem gambling was 1.1%. The gambling factors related with the level of adolescent problem gambling include the presence of nearby gambling facilities, having personal relationships with people that gamble, a higher number of experienced gambling behaviors, male adolescents, and a greater amount of time spent gambling. To the best of our knowledge, this study is the first report to identify gambling factors related with the level of adolescent problem gambling in Korean adolescents using national data. These findings suggest that gambling prevention efforts must consider not only access to individual adolescents as early intervention, but also environmental strategies such as accessibility regulations and alternative activities.

## 1. Introduction

Gambling has come to mean wagering money or other belongings on chance activities or events with random or uncertain outcomes [1,2]. Gambling, a socially acceptable recreational activity, is difficult for adolescents to access because of the legal age limit. Gambling disorder (GD) is defined as a psychiatric state of continuous gambling behavior despite the fact that it negatively affects an individual’s important life functions [3,4]. Despite the fact that many people perceive problem gambling to be an issue prevalent only in adults, recent research indicates that problem and pathological gambling pose serious concerns among adolescents [5,6,7]. The characteristics and repetition of gambling behaviors are also seen in problem gambling of adolescents, and adolescents have a tendency to lose control and to be impulsive [5,8]. Adolescent gambling behavior leads to clinically significant impairment or distress, including criminal behavior, poor academic achievement, school truancy, financial problems, depressive symptoms, suicide, low self-esteem, deterioration of social relationships, and substance abuse [8,9,10,11]. About 70% of adults with a gambling addiction are reported to have started gambling in adolescence in Korea [12]. The true nature of gambling problems in adolescents is the unique clinical nature. In adolescent populations, due to having limited financial resources and being legally restricted by gambling age, the core issues may relate to the large amounts of time being devoted to gambling-related behaviors [13]. In the last four years, crane games (so-called ‘doll drawing games’) have become popular among Korean adolescents. According to the Game Rating and Administration committee in Korea, the number of doll drawing game shops has increased by 81 times in the past two years [14]. Indeed, adolescents dream to become a “king of drawing” or “pro drawer.” In addition, communities and videos are becoming popular, sharing the best place to draw dolls and how to draw dolls well. Adolescents who become the king of drawing or pro drawer carry their dolls in their bags to show off their abilities, or they post related pictures on the SNS (social network service). In addition, they also sell the dolls they draw in the online market to cover the cost of another drawing game. In recent years, competition among doll drawing shops is getting worse, and illegal drawing such as expensive figures and electronic devices are being prized. Even some adolescents were arrested by police officers while stealing a doll by putting themselves directly into the exit gate of the doll drawing machine [15]. The doll drawing games among adolescents is not for simple pleasure since it instigates the individual to gamble. It leads adolescents to experience gambling by spending one’s money on luck and looking forward to receiving money or presents. Due to this point of view, doll drawing games share some similarities with gambling in Korea.

The average adolescent gambling rate was shown to be 57.4% in the past 12 months [5,8]: it ranged from 61.4% to 68.0% in North America, 47.8% to 70.4% in Oceania, and 39.9% to 79.1% in Europe, and was 6.9% in Brazil. The easier the accessibility to gambling among adolescents is, the higher the prevalence is. There are some variations in problem gambling prevalence rates in different regions [6,11,16]: in North America, problem gambling rates ranged from 2.1% to 2.6%; in Oceania, they ranged from 0.2% to 4.4%; and in Europe, they ranged from 0.2% to 12.3%. In Athens, the problem gambling rate was identified to be 5.6% in a sample. In fact, in many studies, the problem gambling rates of adolescents were similar to the adult problem gambling rate [7,10,17]. Therefore, problem gambling by adolescents should not be overlooked. There are various opinions on the causes of problem gambling, such as biological or emotional vulnerability and ecological factors. An adolescent who has repeatedly witnessed the gambling of people around them has a fantasy of winning, considering the behavior of significant others as fun or a game [17,18]. In particular, the onset of gambling behaviors is more likely to start early, before the age of 10 [19,20], and adolescents exposed to gambling are more likely to engage in risky behavior [21], so a preventive approach for adolescents is important. 

So far, several studies have been reported that classify the prevalence of gambling and subtype group characteristics among adolescents in each country. There have been few reports on the gambling characteristics and prevalence of Korean adolescents internationally. Some studies have looked at the level of problem gambling of adolescents mainly in relation to specific variables (e.g., gender, age of first gambling, impulsiveness). Furthermore, since GD has the characteristics of a progressive disorder, there is a limit to the dichotomy of the gambling problem for adolescents. The Canadian Adolescent Gambling Inventory (CAGI) was developed to measure gambling behavior among adolescents based on theoretical and methodological evidence [22,23]. The CAGI provides gambling problems ranging from no problem to a high severity problem and measures gambling-related psychosocial consequences. In order to understand the problem gambling of adolescents, it is necessary to examine the relationship between these gambling factors and the gambling problem level. The purpose of this study was to investigate the gambling factors related with the gambling problem level of adolescents using the CAGI and to provide basic information for the prevention of adolescent gambling problems. 

## 2. Materials and Methods

### 2.1. Study Design

This is a descriptive secondary data analysis study conducted to identify the level of problem gambling of adolescents and gambling factors by utilizing the 2015 National Survey on Youth Gambling Problems. 

### 2.2. Study Subjects

The subjects of this study were 14,011 male and female adolescents from the first grade of a middle school to sophomores in high school who participated in the 2015 National Survey on Youth Gambling Problem conducted by the Korean Center on Gambling Problems. 

### 2.3. Measurements

This study used demographic variables (gender, age, residential area, school year), gambling-related variables (experience and pattern of gambling behaviors, and gambling environments), and the level of problem gambling that are consistent with the purpose of this study. 

#### 2.3.1. Level of Problem Gambling

We used the Canadian Adolescent Gambling Inventory (CAGI), a gambling tool that was developed by Tremblay et al [22], for adolescents aged 13 to 17 years. The gambling problem was assessed using nine of the 24 items, indicating the Gambling Problem Severity Scale (GPSS) during the past three months. The nine items are as follows: skip practice or drop out of activities; skip hanging out with friends; plan gambling/betting activities; feel bad; go back another day to try to win; hide gambling/bets from others; feel that gambling/betting is a problem; take money from lunch/clothing allowance, etc.; and steal money in order to gamble/bet. The GPSS uses a four-point Likert scale (no, 0 point; sometimes (1–3 times), 1 point; often (4–6 times), 2 points; and almost always (≥7 times), 3 points) and yields a score ranging from 0 to 27. The final score gives the degree of global gambling severity, and the scores are classified into three categories: (1) 0–1 no problem gambling (“Green light”), (2) 2–5 low to moderate severity (“Yellow light”), and (3) 6+ high severity (“Red light”). The GPSS provided a good classification accuracy for a cut-off point of 6, with a sensitivity of 0.97 and specificity of 0.93. At the time of development, the Cronbach’s α value was 0.83~0.90. The Korean Center on Gambling Problems acquired a use agreement from the instrument developer for the survey on adolescent problem gambling in 2015, and then gathered validated researchers and examined the reliability/validity of the Korean translation instrument using an online panel of adolescents. The Cronbach’s α value was 0.85 in this study.

#### 2.3.2. Experience and Pattern of Gambling Behaviors

Experience of gambling behaviors includes the number of experienced gambling behaviors; number of direct gambling behaviors in the last three months; frequency of gambling behavior (under 1 time/week, 2–6 times/week, almost every day within three months); spent time on gambling (minutes); average time from the start of gambling to the end of the day within three months; money (KRW) spent on gambling, which means the gambling expenditure within three months; money (KRW) lost by gambling within three months; and money (KRW) won by betting in the past. Additionally, it includes the age of gambling onset, which refers to the average age at which gambling was first initiated among adolescents who have experienced lifetime gambling.

The patterns of gambling behaviors were classified into an on-line type and off-line type, depending on whether the environment in which gambling behavior is exhibited is on-line or off-line. There are six kinds of off-line types of gambling behavior, such as cards or hwatu; claw or prize; sports betting; wagering; national lottery purchases such as lotto, sports toto, and etc.; and betting on horses, bicycles, or motor boats. There are also six kinds of on-line types of gambling behavior, such as cards or hwatu using Hangame (www.hangame.com) or Netmarble (www.netmarble.net) etc., online wagering, online lottery purchases, online sports betting using bet-man, online illegal sports betting, and internet casinos (see Appendix A). These patterns were measured by dividing the time of initial gambling behavior and the time of gambling behavior within three months.

#### 2.3.3. Gambling Environments

The gambling environment includes personal relationships that gamble and the presence of nearby gambling facilities.

The personal relationships that gamble includes family members, friends, and people around the individual who gamble. 

The presence of nearby gambling facilities includes gambling places (or facilities) that are in the administrative residential area and around the house or school (betting shop, race track, bullfight stadium, casino, lottery store, and adult entertainment room). The existence of nearby gambling facilities in the residential area seems to be due to the tendency of visiting gambling places in residential areas for adolescents who are already experiencing problem gambling, not the simple curiosity of those adolescents.

### 2.4. Data Collection

The population of original data was 1,717,911 middle school students and 1,839,732 high school students among the statistics provided by the Educational Statistics Service [24] as of April 2014. A square root proportional distribution was used to prevent the sample from having a sampling tipping effect by region (proportionally allocating the sample size by region to the population ratio leads to excessive sampling in the Seoul, Gyeonggi, Busan, and Kyungnam areas) in the sampling design stage. In the case of schools with more than 25 students per class, the probability proportional system method was used for the sample selection according to the type of school sector, type of school, and coeducation. The primary stratification variable for the survey was the area where the school was located. The selected schools were 120 middle schools and 130 high schools. With the cooperation of metropolitan and provincial offices of education, two grades were examined in one school, one in each of two grades. The schools that rejected the survey were replaced with pilot sample schools that were previously extracted from the same stratification. Post-stratification and weighting were applied to match the distribution of the samples and the populations after the survey. 

The original data collection was conducted from 21 August to 3 October 2015. Experienced and trained interviewers visited each classroom in the school, and data were collected in the form of a self-report questionnaire. Before conducting the survey, the interviewer first asked the teacher and manager to leave the classroom. The interviewer then explained to the subject the contents related to ethical matters (the purpose and time of the investigation, the risks and benefits to the participant, the confidentiality of the data, the restriction of accessibility, and autonomous participation) and received the written consent of the subject in the study. 

The personal information of the subject was deleted from the original data for the data used in this study. In this way, the anonymity and confidentiality of the subject were guaranteed, and the research was approved by the Institutional Review Board (IRB) of Konkuk University in terms of ethical consideration of the study (No. 7001355-201708-E-051). 

### 2.5. Data Analysis

All statistical analyses were conducted using the IBM SPSS 23.0 version for Windows program after minimizing sampling error. Using a specialized punching program called Survey Craft, we completely blocked errors that did not fit the logic or input errors of punchers. In addition, through edits, we confirmed errors or omissions and logical inconsistencies in records, and discarded problem tables. The punching data was subjected to data cleaning in accordance with items such as a response base, non-response, extreme value, logical error, etc., in the computer processing department. The data that were cleaned by data were generated by using the main variables, and cross tabulation was reviewed in the research.

Categorical data were calculated using the frequency and percentage, and continuous data using the mean and standard deviation. The sociodemographic characteristics and gambling behavior-related characteristics were analyzed by Chi-square’s test, Fisher’s exact test, and one-way ANOVA, and then post-hoc by Scheffe’s test. The gambling factors related with the level of gambling problem were verified with multinomial logistic regression by maximum likelihood estimation. The Green light (no problem) was set as the baseline category (as reference), and Yellow light (low to moderate severity) and Red light (high severity) as the comparative categories. Hosmer and Lemeshow tests were performed to evaluate the goodness of fit for the logistic regression analysis. The explanatory power of the model was estimated by the coefficient of determination of Nagelkerke. The probability of problem gambling was calculated as the odds ratio (OR) when the gambling-related factors are exposed. The OR is nonnegative, with a value greater than 1.0, when success is more likely than failure [25]. Selection of the final model depended on the achievement of statistical significance (*p* < 0.05).

## 3. Results

### 3.1. General Characteristics

The survey respondents were 52.5% males and 47.5% females. The average age was 14.9 ± 1.4 years. The residential areas were 16.8% in the capital (Seoul), 26.1% in metropolitan cities, and 57.0% in provinces. The age of gambling onset was 12.1 ± 2.7 years. The lifetime gambling behavior experience was 42.1%, and 24.2% had a gambling behavior experience within the past three months. The prevalence of the gambling problem level through the GPSS/CAGI was classified as 94.9% for no problem (Green light), 4.0% for a low to moderate severity level (Yellow light), and 1.1% for a high severity level (Red light) (Table 1).

### 3.2. Characteristics of Gambling According to the Level of Problem Gambling

The variables that showed significant differences according to the level of problem gambling are as follows: pattern of initial gambling behavior (x^2^ = 143.758, *p* < 0.001), pattern of gambling behavior within three months (x^2^ = 151.778, *p* < 0.001), frequency of gambling behavior (x^2^ = 236.623, *p* < 0.001), number of experienced gambling behaviors (F = 2082.636, *p* < 0.001), time spent gambling (F = 64.789, *p* < 0.001), money used on gambling (F = 117.235, *p* < 0.001), money lost by gambling (F = 40.755, *p* < 0.001), money won by betting (F = 56.934, *p* < 0.001), gambling of personal relationships (x^2^ = 479.788, *p* < 0.001), and nearby gambling facilities (x^2^ = 62.896, *p* < 0.001). In addition, there were significant differences in gender (x^2^ = 66.826, *p* < 0.001) and age (F = 3.413, *p* = 0.033), among general characteristics (Table 2).

### 3.3. Gambling Factors related with the Level of Adolescent Problem Gambling

As a result of testing the multicollinearity for the gambling factors related with the level of adolescent problem gambling, the tolerance limit was 0.625~0.918, which was below 1.0. Additionally, the variance inflation factor was 1.089–1.600, which was not more than 10, indicating that there was no problem of multicollinearity. To analyze the gambling factors related with the level of adolescent problem gambling, multivariate logistic regression analysis was conducted by classifying gambling levels as a no problem gambling level (Green light), low to moderate severity gambling level (Yellow light), and high severity gambling level (Red light). The explanatory power of the final model was statistically significant at Nagelkerke R^2^, 0.241(−2 log likelihood, 2319.14, *p* < 0.001). 

The results of the gambling factors at the low to moderate severity gambling level (Yellow light) were as follows: the participants were significantly more likely to have personal relationships that gambled (OR = 1.534, 95% CI 1.189–1.978), to have a greater number of experienced gambling behaviors (OR = 1.256, 95% CI 1.163–1.357), and to exhibit more time spent gambling (OR = 1.004, 95% CI 1.002–1.007). When the age of gambling onset increased by one unit, it was less likely to increase 0.936 times (*p* = 0.003). 

Gambling factors at the high severity gambling level (Red light) were as follows: the participants were significantly more likely to have nearby gambling facilities (OR = 2.151, 95% CI 1.263–3.660), to have personal relationships that gambled (OR = 1.999, 95% CI 1.254–3.188), to be male adolescents (OR = 1.952, 95% CI 1.133–3.365), and to exhibit more time spent on gambling (OR = 1.005, 95% CI 1.001–1.008). When the age of gambling onset increased by one unit, it was less likely to increase 0.871 times (*p* < 0.001) (Table 3).

## 4. Discussion

This study included 14,011 male and female adolescents who participated in the 2015 National Survey on Youth Gambling Problem conducted by the Korean Center on Gambling Problems.

The main discussions related to the results of this study are as follows.

First, throughout this study, the prevalence of lifetime gambling behavior experience was 42.1%. The problem gambling level determined through the GPSS/CAGI was classified as 94.9% for no problem (Green light), 4.0% for a low to moderate severity level (Yellow light), and 1.1% for a high severity level (Red light). For the reason of volume, this study did not show all of the specific types of gambling behavior patterns, but the specific types of gambling behavior patterns are presented separately as an appendix to help readers understand the results (see Appendix A). As a result of this study, according to the no problem gambling level (Green light) which accounted for 94.9% of participants, the gambling type that showed the highest frequency was crane games. In recent years, crane games (so called ‘doll drawing games’) have become popular among Korean adolescents. As the number of doll drawing game shops has increased greatly, and doll drawing machines are mainly located at street and convenience stores, this has made it easy for adolescents to access them [14,15]. The problem is that the social controversy is spreading because adolescents are excessively engaged in crane games and spend a lot of time and money in order to acquire expensive electronics, as well as dolls [15]. Nevertheless, the majority of adolescents and adults do not recognize crane games as gambling. Not surprisingly, online illegal sports betting and online casino gambling are also happening in the no problem gambling level (Green light). In other words, the no problem gambling level (Green light) has not reached the level of problem gambling at present, but the easier and more accessible gambling behavior can be used to consider that the possibility of problem gambling might be inherent in the future. Therefore, it is very important to prevent gambling behaviors of adolescents in advance. 

Second, compared to the no problem gambling level (Green light), having personal relationships that gamble (the presence of family members, friends, and people around the individual who gamble) was a gambling factor for adolescent problem gambling in both the Yellow light (level of low to moderate severity) and Red light (level of high severity). This finding is consistent with the results of previous studies, indicating that the risk of gambling behaviors among adolescents increases when they have friends and people around the individual who gamble, as well as when a greater number of their parents or relatives are engaged in gambling behavior. Furthermore, these adolescents tend to accept the gambling problem more spontaneously [23,26,27]. In addition, compared with the no problem gambling level (Green light), the presence of nearby gambling facilities in residential areas was the most related factor for adolescent problem gambling in the Red light (level of high severity). These results showed that gambling accessibility is an important factor in ecological attributes. Thus, people who live where gambling is legal can easily access gambling, and adolescents living in these areas easily accept gambling behaviors and become insensitive to gambling problems [28]. In the case of regulating adolescents’ gambling access, the adolescent problem gambling prevalence rate decreased to 1.3–1.6% in Brazil and Denmark, while the adolescent problem gambling prevalence rate increased to 5.3% in Albania without adolescent gambling access regulation. These results suggest that an environmental strategy such as accessibility regulations, as well as individual environmental approaches for adolescents, is necessary. Therefore, in order to prevent adolescent gambling addiction in the future, it is necessary not only to fulfill social responsibilities such as regulations on gambling establishments and access, but also to emphasize strategies for finding various alternatives, such as play and culture, that can promote adolescent participation. 

Third, in this study, male adolescents showed a significant presence in the high severity gambling level (Red light) when compared with the no problem gambling level (Green light). This is consistent with the results of a meta-analytic study in which the relationship between male adolescents and problem gambling was high [19] and there was a high tendency toward addiction in male adolescents [20,23,29]. Male adolescents show a weakness that is difficult to control if they indulge in gambling once [30], and they are more interested in competition and victory [10]. In addition, the pattern of gambling behavior preferred by male adolescents, such as card games, casinos, lottery purchases, and online gambling, seems to have affected the gambling problem level. Particularly, the pattern of gambling behaviors of male adolescents was found to affect the low to moderate gambling severity level (Yellow light) [31,32]. Therefore, it is necessary to investigate the causal relationship according to gender as a factor relating to the level of adolescent problem gambling in the future, and evidence-based prevention activities reflecting these factors are necessary. 

Fourth, the spent time on gambling in the high severity gambling level (Red light) and the number of experienced gambling behaviors in the low to moderate gambling severity level (Yellow light) have a significant impact. Compared to the spent time on gambling at the no problem gambling level (Green light), the spent time on gambling behavior in both the low to moderate severity gambling level (Yellow light) and high severity gambling level (Red light) was longer than 20–57 min, and the money used on gambling was more than 20,000–215,000 KRW. These results showed that habitual gambling is highly related to the adolescent problem gambling in ecological and biological attributes. The results of this study confirm that the time and money spent on gambling behaviors are factors that increase the level of adolescent problem gambling, which is consistent with previous research results [31]. A vicious cycle where the addiction problem becomes more severe is repeated [33,34], because adolescents with a high severity gambling level (Red light) spend more time on various patterns of gambling behaviors to make up for lost money [35]. In particular, adolescents who gamble at the problem level are seeking the dramatic fun of winning, so they are continuously gambling for a stronger stimulus. Due to persistent gambling behaviors, a variety of problems such as criminal behavior, poor academic achievement, school truancy, financial problems, depression, suicide, deterioration of social relationships, and substance abuse, have arisen [8,9,10,11]. These problems are diagnostic criteria for clinical gambling disorders in DSM-5 [36]. However, adolescents who gamble showed a lack of understanding of the negative consequences of gambling and did not fully appreciate the term ‘quit’. Additionally, various gambling-related advertisements promote the interest of gambling and fun of winning for adolescents [9]. In order to prevent the gambling addiction of adolescents, it is necessary to intensively educate them on economic ethics, such as the time and money spent on gambling behaviors, and help adolescents to make responsible choices. In addition, with active parent education, parents should supervise their adolescents to lower the acceptability and availability of the gambling, because the insufficient coping skills of adolescents can lead to problem gambling [19,37,38,39]. In schools in particular, adolescents who already have problems with gambling should be exposed to active intervention early so that they do not progress to gambling addiction. 

Lastly, the age of gambling onset showed a relatively low relevance in gambling factors. The age of gambling onset refers to the average age at which gambling was first initiated among adolescents who have experienced lifetime gambling. This study showed that the age of gambling onset was 12.1 ± 2.1 years old; that is, gambling behavior started around elementary school grades 5 and 6. This is similar to the results of an overseas study which stated that the age of gambling onset was 10–11 years old [19,20]. Since the age of gambling onset is reported to be a risk factor for future gambling addiction [19], it is important to keep in mind that adolescents who start gambling at a young age are likely to progress to high severity gambling levels [29,39]. As society grows, there are more opportunities to access gambling, and the patterns of gambling behaviors are changing in the form of online gambling. Therefore, the age of gambling onset is very important because the accessibility of gambling for adolescents will be further increased. The fact that teenagers engage in gambling behaviors means that adolescents can use gambling as a means of escaping from their situation, and negative consequences such as derailment or school maladjustment may become serious [40]. This suggests that gambling prevention activities should be concentrated on adolescents because gambling can make it difficult for them to reach their required academic achievement, and it may hinder the formation of a desirable personality and values. Therefore, it is necessary to convey general information including protective factors and risk factors of gambling behaviors to adolescents aged 10–12, and to educate them about mathematical concepts (e.g., odds and house edge) related to the probability of winning a lottery or randomness to help them make healthy decisions [39]. 

The significance of this study is as follows. First, the representativeness of data was secured by studying all the adolescents in Korea in 2015. Second, gambling factors related with the level of adolescent problem gambling were identified. Third, we suggested a strategy to prevent adolescent problem gambling. Despite these implications, this study has some limitations, including responder biases and reliance on self-reporting, potentially leading to the inaccurate recall of past behaviors. Given the often illegal nature of several variables of interest, it is also possible that the participants may have deliberately failed to report certain activities. The other limitation of this study is that the measurement of cognitive and psychological factors was insufficient compared to the gambling-related factors. In this study, we propose some suggestions. First, further investigation of cognitive and psychological factors is needed to more comprehensively understand the gambling factors. We also suggest an exploratory study to confirm the causal relationship between gambling factors related with adolescent problem gambler.

## 5. Conclusions

In this study, the prevalence of lifetime gambling behavior experience was 42.1%, and 24.2% had a gambling experience within the past three months. The past three-month prevalence of problem gambling was 1.1%. Additionally, the age of gambling onset was 12 years old. The level of problem gambling was related to environmental factors such as the presence of nearby gambling facilities and having personal relationships with people that gambled. In addition, the factors of greater number of gambling behaviors experienced, male adolescents, and a great amount of time spent gambling, were more likely to lead to the possibility of problem gambling. 

To the best of our knowledge, this study is the first report to identify gambling factors related with the level of problem gambling in Korean adolescents using national data. 

Considering the age of gambling onset (12 years old), the gambling experience rate within the past three months (24.2%), and the past three-month prevalence of problem gambling (1.1%) in this study, early intervention strategies to prevent adolescent problem gambling are needed. In addition, gambling prevention efforts must consider environmental strategies such as accessibility regulations and alternative activities. Ultimately, this study is expected to increase interest in the prevention of adolescent problem gambling and to develop effective preventive measures for adolescents.

## Figures and Tables

**Table 1:** General characteristics of the study population.

Item	Frequency	%	Mean ± SD
**Total**	14,011	100.0	
**Gender**			
Female	6662	47.5	
Male	7349	52.5	
**Age**			14.9 ± 1.4
**School year**			
Middle school 1	2346	16.7	
Middle school 2	2670	19.1	
Middle school 3	3020	21.6	
High school 1	2974	21.2	
High school 2	3001	21.4	
**Residential areas**			
Capital (Seoul)	2357	16.8	
Metropolitan	3664	26.1	
Province	7990	57.1	
**Lifetime gambling behavior experience**			
No	8116	57.9	
Yes	5895	42.1	
**Age of gambling onset (year)** **^a^**			12.1 ± 2.7
**Gambling behavior experience within the past 3 months**			
No	10,618	75.8	
Yes	3393	24.2	
**Number of experienced gambling behaviors ^b^**			0.5 ± 1.2
**GPSS/CAGI score**			
≤1 (Green light, no problem)	13,297	94.9	
2–5 (Yellow light, low to moderate severity)	563	4.0	
≥6 (Red light, high severity)	152	1.1	
**Gambling of personal relationships ^c^**			
No or Do not know	11,795	84.2	
Yes	2216	15.8	
**Nearby gambling facilities ^d^**			
No or Do not know	12,888	92.0	
Yes	1123	8.0	

^a^ Age of gambling onset: the average age at which gambling was first initiated among adolescents who have experienced lifetime gambling; ^b^ Number of experienced gambling behaviors: number of direct gambling behaviors in the last three months; ^c^ Gambling of personal relationships: the presence of family members, friends, and people around the individual who gamble; ^d^ Nearby gambling facilities: gambling places (or facilities) that are in the administrative residential area and around the house or school (betting shop, race track, bullfight stadium, casino, lottery store, and adult entertainment room).

**Table 2:** Characteristics of gambling according to the level of problem gambling.

Item	Green Light *n*(%) or Mean ± SD	Yellow Light *n*(%) or Mean ± SD	Red Light *n*(%) or Mean ± SD	Overall (*n* = 14,001, 100%)	χ^2^/F	*p*
**Total**	13,297 (94.9)	562 (4.0)	152 (1.1)	14,011 (100.0)	66.826	<0.001
**Gender**						
Female	6419 (96.4)	210 (3.1)	33 (0.5)	6662 (47.5)		
Male	6878 (93.6)	352 (4.8)	119 (1.6)	7349 (52.5)		
**Age (year)**	15.1 ± 1.5 ^(a)^	15.1 ± 1.4 ^(a)^	15.4 ± 1.3 ^(b)^		3.413	0.033
**Age of gambling onset (year) ^a^**	12.5 ± 2.8	12.3 ± 2.8	12.5 ± 3.2		0.808	0.446
**Pattern of initial gambling behavior ^b^**					143.758	<0.001
Off-line	4951 (89.0)	503 ( 9.0)	114 ( 2.0)	5568 (94.5)		
On-line	230 (70.3)	60 (18.4)	37 (11.3)	327 ( 5.5)		
**Pattern of gambling behavior within 3 months ^b^**					151.778	<0.001
Off-line	2523 (80.9)	493 (15.8)	103 ( 3.3)	3119 (91.9)		
On-line	156 (56.7)	70 (25.5)	49 (17.8)	275 ( 8.1)		
**Frequency of gambling behavior ^c^**					236.623 *	<0.001
≤1 time per week	2552 (81.2)	488 (15.5)	101 ( 3.3)	3141 (92.6)		
2–6 times per week	115 (53.5)	63 (29.3)	37 (17.2)	215 ( 6.3)		
Almost every day	12 (31.6)	12 (31.6)	14 (36.8)	38 ( 1.1)		
**Number of experienced gambling behaviors ^d^**	0.4 ± 1.1 ^(a)^	2.8 ± 1.8 ^(b)^	3.8 ± 2.4 ^(c)^		2082.636	<0.001
**Spent time on gambling (minutes) ^e^**	37.0 ± 48.3 ^(a)^	57.2 ± 68.6 ^(b)^	94.7 ± 119.2 ^(c)^		64.789	<0.001
**Used money on gambling (KRW) ^f^**	8864.7 ± 23,076.1 ^(a)^	29,702.8 ± 78,242.7 ^(a)^	223,393.6 ± 59,746.5 ^(b)^		117.235	<0.001
**Lost money by gambling (KRW) ^g^**	4970.9 ± 16,169.9 ^(a)^	13,502.3 ± 40,165.9 ^(a)^	88,966.4 ± 27,650.4 ^(b)^		40.755	<0.001
**Won money by betting (KRW) ^h^**	17,143.3 ± 72,880.5 ^(a)^	40,273.1 ± 25,898.4 ^(a)^	134,904.4 ± 41,707.9 ^(b)^		56.934	<0.001
**Residential areas**					15.120	0.004
Capital (Seoul)	2270 (96.2)	75 (3.2)	13 (0.6)	2358 (16.9)		
Metropolitan	3483 (95.1)	141 (3.8)	39 (1.1)	3663 (26.1)		
Province	7544 (94.4)	347 (4.3)	99 (1.3)	7990 (57.0)		
**Gambling of personal relationships ^i^**					479.788	<0.001
Yes	1899 (85.7)	232 (10.5)	85 (3.8)	2216 (15.8)		
No or Do not know	11,398 (96.6)	331 ( 2.8)	67 (0.6)	11,796 (84.2)		
**Nearby gambling facilities ^j^**					62.896	<0.001
Yes	1017 (90.6)	72 (6.4)	34 (3.0)	1123 ( 8.0)		
No or Do not know	12,280 (95.3)	491 (3.8)	117 (0.9)	12,888 (92.0)		

* Fisher’s exact test. ^(a)–(c)^ Scheffe’s test (mean with the other letter significantly different). ^a^ Age of gambling onset: the average age at which gambling was first initiated among adolescents who have experienced lifetime gambling; ^b^ Pattern of gambling behavior: divided by the time of initial gambling behavior and the time of gambling behavior within three months. ^c^ Frequency of gambling behavior: under 1 time/week, 2–6 times/week, and almost every day within three months; ^d^ Number experienced gambling behaviors: number of direct gambling behaviors in the last three months; ^e^ Time spent on gambling (minutes): average time from the start of gambling to the end of the day within three months; ^f^ Money used on gambling (KRW): the gambling expenditure within three months; ^g^ Money lost by gambling (KRW): money lost by gambling within three months. ^h^ Money won by betting (KRW): money won by betting in the past; ^i^ Gambling of personal relationships: the presence of family members, friends, and people around the individual who gamble; ^j^ Nearby gambling facilities: gambling places (or facilities) that are in the administrative residential area and around the house or school (betting shop, race track, bullfight stadium, casino, lottery store, and adult entertainment room).

**Table 3:** Multinomial regression for gambling factors related with the level of adolescent problem gambling.

Variables	Model (Reference: Green Light)					
	Yellow Light			Red Light		
	Odds Ratio	95% CI *	*p*	Odds Ratio	95% CI	*p*
**Gender**						
Male	1.007	0.780–1.299	0.958	1.952	1.133–3.365	0.016
Female (Reference)						
**Residential areas**						
Province	0.946	0.612–1.352	0.759	0.450	0.199–1.015	0.054
Metropolitan	0.897	0.674–1.193	0.454	0.920	0.557–1.520	0.746
Capital (Reference)						
**Gambling of personal relationships ^a^**						
Yes	1.534	1.189–1.978	<0.001	1.999	1.254–3.188	0.004
No or Do not know (Reference)						
**Nearby gambling facilities ^b^**						
Yes	1.122	0.782–1.610	0.533	2.151	1.263–3.660	0.005
No or Do not know (Reference)						
**Age of gambling onset (year) ^c^**	0.936	0.896–0.977	0.003	0.871	0.807–0.941	<0.001
**Number of experienced gambling behaviors ^d^**	1.256	1.163–1.357	<0.001	1.328	1.185–1.489	<0.001
**Pattern of gambling behavior within 3 months ^e^**						
On-line	1.109	0.688–1.788	0.670	0.666	0.347–1.276	0.220
Off-line (Reference)						
**Frequency of gambling behavior ^f^**						
Almost every day	1.005	0.325–3.110	0.994	0.517	0.132–2.023	0.340
2–6 times per week	1.617	0.495–5.286	0.420	1.577	0.384–6.483	0.490
≤1 time per week (Reference)						
**Spent time on gambling (minutes) ^g^**	1.004	1.002–1.007	0.002	1.005	1.001–1.008	0.004
**Used money on gambling (KRW) ^h^**	1.000		<0.001	1.000		<0.001
**Lost money by gambling (KRW) ^i^**	1.000		0.780	1.000		0.509
**Won money by betting (KRW) ^j^**	1.000		0.950	1.000		0.679
−2 log likelihood	2319.14					
Chi-square (df)	377.86 (28)					
Nagelkerke R2	0.241					

* CI: Confidence Interval. ^a^ Gambling of personal relationships: the presence of family members, friends, and people around the individual who gamble; ^b^ Nearby gambling facilities: gambling places (or facilities) that are in the administrative residential area and around the house or school (betting shop, race track, bullfight stadium, casino, lottery store, and adult entertainment room); ^c^ Age of gambling onset: the average age at which gambling was first initiated among adolescents who have experienced lifetime gambling; ^d^ Number of experienced gambling behaviors: number of direct gambling behaviors in the last three months; ^e^ Pattern of gambling behavior: divided by the time of initial gambling behavior and the time of gambling behavior within three months; ^f^ Frequency of gambling behavior: under 1 time/week, 2–6 times/week, and almost every day within three months; ^g^ Time spent on gambling (minutes): average time from the start of gambling to the end of the day within three months; ^h^ Money used on gambling (KRW): the gambling expenditure within three months; ^i^ Money lost by gambling (KRW): money lost by gambling within three months; ^j^ Money won by betting (KRW): money won by betting in the past.

## Appendix A

**Table A1:** Patterns of gambling behavior.

Patterns of Gambling Behaviors	Green Light *n* *(%) ^‡^	Yellow Light *n* *(%) ^‡^	Red Light *n* *(%) ^‡^
**Off-line type**			
Cards or hwatu	397 (11.7)	109 (3.2)	29 (0.9)
Crane game ^a^	1,387 (40.8)	192 (5.7)	32 (0.9)
Sports betting	377 (11.1)	90 (2.6)	23 (0.7)
Wagering (Jjaljjari ^b^, ladder, bingo, etc.)	334 ( 9.8)	96 (2.8)	19 (0.6)
National lotteries purchase (such as lotto, sports toto, etc.)	27 (0.8)	5 (0.1)	0 (0.0)
Betting on horse, bicycle or motor boat	1 (0.0)	2 (0.1)	0 (0.0)
**On-line type**			
Cards or hwatu using Hangame (www.hangame.com), Netmarble (www.netmarble.net) etc.	84 (2.5)	25 (0.7)	4 (0.1)
Online wagering (snail bob, ladder, bingo, etc.)	50 (1.5)	28 (0.8)	31 (0.9)
On-line lotteries purchase	3 (0.1)	4 (0.1)	0 (0.0)
Online sports betting using bet-man	4 (0.1)	0 (0.0)	6 (0.2)
Online illegal sports betting	11 (0.3)	9 (0.3)	8 (0.2)
Internet casino	6 (0.2)	4 (0.1)	0 (0.0)

* The number of cases refers to the number of games to which the respondent answered “yes” in relation to their experience of having participated in each game during the past three months. ^‡^ The percentage of each pattern of gambling behavior in all cases is presented as %; ^a^ Crane games: doll or prize drawing game; ^b^ Jjaljjari: the opponent hides the coin in the first step and lets you guess whether the coin in the first step is even or odd. At this time, you want to bet as much money as you can and get money.

## References

[1] US National Research Council (1999). Pathological Gambling: A Critical Review.

[2] Williams R.J., Volberg R.A., Stevens R.M.G., Williams L.A., Arthur J.N. (2017). Report prepared for the Canadian Consortium for Gambling Research. The Definition, Dimensionalization, and Assessment of Gambling Participation.

[3] American Psychiatric Association [APA] (2013). Diagnostic and Statistical Manual of Mental Disorders.

[4] Ferrara P., Franceschini G., Corsello G. (2018). Gambling disorder in adolescents: What do we know about this social problem and its consequences?. Ital. J. Pediatr..

[5] Calado F., Alexandre J., Griffiths M.D. (2017). Prevalence of adolescent problem gambling: A systematic review of recent research. J. Gambl. Stud..

[6] Hardoon K., Derevensky J.L., Gupta R.J. (2003). Empirical measures vs. perceived gambling severity among youth: Why adolescent problem gamblers fail to seek treatment. Addict. Behav..

[7] Korean Center on Gambling Problems: 2015 National Survey on Youth Gambling Problem. https://www.kcgp.or.kr/np/recsroom/2/recsroomSearch.do.

[8] Volberg R.A., Gupta R., Griffiths M.D., Olason D.T., Delfabbro P. (2010). An international perspective on youth gambling prevalence studies. Int. J. Adolesc. Med. Health.

[9] Hume M., Mort G.S. (2011). Fun, friend, or foe: Youth perceptions and definitions of online gambling. Soc. Mar. Q..

[10] Lynch W.J., Maciejewski P.K., Potenza M.N. (2004). Psychiatric correlates of gambling in adolescents and young adults grouped by age at gambling onset. Arch. Gen. Psychiatry.

[11] Gambling Research Exchange Ontario Driving Knowledge into Action. https://www.greo.ca/Modules/EvidenceCentre/Details/youth-gambling-critical-review-public-health-literature.

[12] Min K.R., Kyo J.K., Sun J.K. (2007). Psychosocial characteristics of internet gamblers in South Korea. Korean J. Health Psychol..

[13] Parker J.D., Taylor R.N., Eastabrook J.M., Schell S.L., Wood L.M. (2008). Problem gambling in adolescence: Relationships with internet misuse, gaming abuse and emotional intelligence. Personal. Individ. Differ..

[14] The Game Rating & Administration Committee. https://www.grac.or.kr/.

[15] Shin H.J., Kim J.C. (2017). Major issues and tasks for gambling disorder in adolescents-focusing on a controversy over speculation in doll drawing game. Korean Assoc. Addict. Crime Rev..

[16] Anagnostopoulos D.C., Lazaratou H., Paleologou M.P., Peppou L.E., Economou M., Malliori M., Papadimitriou G.N., Papageorgiou C. (2017). Adolescent gambling in greater Athens area: A cross-sectional study. Soc. Psychiatry Psychiatr. Epidemiol..

[17] Ha M.J., Park S.Y. (2015). A Study about Youth Gambling Addiction Process: Based on Verbal Statements That Adults Experienced Gambling Addiction During Adolescence. Ment. Health Soc. Work.

[18] Kim S.Y., Kim Y.S., Jeong C.G. (2018). Phenomenological Study on Experience of Adolescent Gambling Behavior. Korean J. Couns..

[19] Dowling N., Merkouris S., Greenwood C., Oldenhof E., Toumbourou J., Youssef G.J. (2017). Early risk and protective factors for problem gambling: A systematic review and meta-analysis of longitudinal studies. Clin. Psychol. Rev..

[20] Lee J.K., Lee R.H., Chang H.L. (2018). Comparisons by gender and school type in the association between betting games and problem gambling among adolescents. J. Digit. Converg..

[21] Shaffer H.J., Hall M.N., Vander Bilt J.J. (1999). Estimating the prevalence of disordered gambling behavior in the United States and Canada: A research synthesis. Am. J. Public Health.

[22] Tremblay J., Stinchfield R., Wiebe J., Wynne H. (2010). Canadian Adolescent Gambling Inventory (CAGI) Phase III Final Report. http://www.ccgr.ca/en/projects/resources/CAGI-Phase-III-Report-English.pdf.

[23] Cho H.C., Park K.W. (2017). A comparative study of different age groups about the effects of ecological systematic factors on the onset of adolescents’ gambling behavior: Applying discrete-time survival analysis. Korean J. Youth Stud..

[24] Korean Educational Statistics Service. http://kess.kedi.re.kr/index.

[25] Agresti A. (2007). An Introduction to Categorical Data Analysis.

[26] Reith G., Dobbie F.J. (2011). Beginning gambling: The role of social networks and environment. Addict. Res. Theory.

[27] Zhai Z.W., Yip S.W., Steinberg M.A., Wampler J., Hoff R.A., Krishnan-Sarin S., Potenza M.N. (2017). Relationships between perceived family gambling and peer gambling and adolescent problem gambling and binge-drinking. J. Gambl. Stud..

[28] Yeon Y.M. (2006). The Influences of Impulsivity, Family’ Gambling Activity and the Distance from Adolescence’s home to the Gambling Facilities Area on adolescents’ gambling activity, gambling belief and gambling motivation in the future. Korean Psychol. J. Cult. Soc. Issues.

[29] Chang J.Y. (2011). Types of gambling and factors influencing adolescent gambling Behavior. Ment. Health Soc. Work.

[30] Kim S.Y., Yoo H.J., Cho I.H., Yune S.K., Lyoo I.K., Ha J.H. (2003). Temperament and Character Profiles of Internet Addiction in Children. Korean J. Psychopathol..

[31] Welte J.W., Barnes G.M., Tidwell M.-C.O., Hoffman J.H. (2009). The association of form of gambling with problem gambling among American youth. Psychol. Addict. Behav..

[32] Mazar A., Williams R.J., Stanek E.J., Zorn M., Volberg R.A. (2018). The importance of friends and family to recreational gambling, at-risk gambling, and problem gambling. BMC Public Health.

[33] Vachon D.D., Bagby R.M. (2009). Pathological gambling subtypes. Psychol. Assess..

[34] Shead N.W., Derevensky J.L., Gupta R. (2010). Risk and protective factors associated with youth problem gambling. Int. J. Adolesc. Med. Health.

[35] Welte J.W., Barnes G.M., Wieczorek W.F., Tidwell M.-C.O., Hoffman J.H. (2007). Type of gambling and availability as risk factors for problem gambling: A Tobit regression analysis by age and gender. Int. Gambl. Stud..

[36] Roehr B. (2013). American Psychiatric Association explains DSM-5. BMJ.

[37] (2017). Korea Center on Gambling Problems: Development of Youth Gambling Prevention Model Based on Gambling Problem and Risk Factors. https://www.kcgp.or.kr/np/recsroom/2/recsroomSearch.do.

[38] Kang K.H., Kim H.S., Park A.R., Kim H.Y., Lee K.S. (2018). Latent Class Analysis of Gambling Activities among Korean Adolescents. J. Korean Acad. Nurs..

[39] Canale N., Vieno A., Ter Bogt T., Pastore M., Siciliano V., Molinaro S. (2016). Adolescent Gambling-Oriented Attitudes Mediate the Relationship Between Perceived Parental Knowledge and Adolescent Gambling: Implications for Prevention. Prev. Sci..

[40] Storr C.L., Lee G.P., Derevensky J.L., Ialongo N.S., Martins S.S. (2012). Gambling and adverse life events among urban adolescents. J. Gambl. Stud..

